# Antimicrobial potential of a ponericin-like peptide isolated from *Bombyx mori* L. hemolymph in response to *Pseudomonas aeruginosa* infection

**DOI:** 10.1038/s41598-022-19450-8

**Published:** 2022-09-15

**Authors:** Jannatun Nesa, Swapan Kumar Jana, Abdul Sadat, Kinkar Biswas, Ahmet Kati, Ozge Kaya, Rittick Mondal, Paulami Dam, Mintu Thakur, Anoop Kumar, Maidul Hossain, Lucas R. Lima, Samilla B. Rezende, Debjoy Bhattacharjya, Debnirmalya Gangopadhyay, Suvankar Ghorai, Sevde Altuntas, Amiya Kumar Panda, Pinak Chakrabarti, Shambhu Swarnakar, Joydeep Chakraborty, Berfin Yilmaz, Maria L. R. Macedo, Octávio L. Franco, Marlon H. Cardoso, Amit Kumar Mandal

**Affiliations:** 1 Chemical Biology Laboratory, Department of Sericulture, Raiganj University, North Dinajpur, 733134 India; 2 Department of Biochemisty, Bose Institute, P1/12 CIT Scheme, VIIM, Kolkata, 700054 India; 3 Insect Ecology and Conservation Biology Laboratory, Department of Sericulture, Raiganj University, North Dinajpur, 733134 India; 4 Laboratory of Organic Synthesis, Department of Chemistry, Raiganj University, North Dinajpur, 733134 India; 5 Experimental Medicine Research and Application Center, University of Health Sciences, Validebag Research Park, Uskudar, 34662 Istanbul, Turkey; 6 TERRA Analysis and Measurement Device Tic. Inc., Istanbul, Turkey; 7 ANMOL Laboratory, Department of Biotechnology, North Bengal University, Darjeeling, 734013 India; 8 Department of Chemistry and Chemical Technology, Vidyasagar University, Midnapore, 721102 India; 9 S-Inova Biotech, Programa de Pós-graduação em Biotecnologia, Universidade Católica Dom Bosco (UCDB), Campo Grande, 79117900 Brazil; 10 Silkworm Rearing Technology and Extension Laboratory, Department of Sericulture, Raiganj University, North Dinajpur, West Bengal 733134 India; 11 Silkworm Genetics and Breeding Laboratory, Department of Sericulture, Raiganj University, North Dinajpur, 733134 India; 12 Department of Microbiology, Raiganj University, North Dinajpur, 733134 India; 13 Department of Botany, Raiganj University, North Dinajpur, 733134 India; 14 Laboratório de Purificação de Proteínas e suas Funções Biológicas, Universidade Federal de Mato Grosso do Sul (UFMS), Cidade Universitária, Campo Grande, Mato Grosso Do Sul 79070900 Brazil; 15 Centro de Análises Proteômicas e Bioquímicas, Programa de Pós-graduação em Ciências Genômicas e Biotecnologia, UniversidadeCatólica de Brasília (UCB), Brasília, 70790160 Brazil; 16 Centre for Nanotehnology Sciences (CeNS), Raiganj University, North Dinajpur, 733134 India

**Keywords:** Biological techniques, Chemical biology, Microbiology

## Abstract

The main effectors in the innate immune system of *Bombyx mori* L. are antimicrobial peptides (AMPs). Here, we infected *B. mori* with varied inoculum sizes of *Pseudomonas aeruginosa* ATCC 25668 cells to investigate changes in morpho-anatomical responses, physiological processes and AMP production. Ultraviolet–visible spectra revealed a sharp change in λ_max_ from 278 to 285 nm (bathochromic shift) in the hemolymph of infected *B. mori* incubated for 24 h. Further, Fourier Transform InfraRed studies on the hemolymph extracted from the infected *B. mori* showed a peak at 1550 cm^−1^, indicating the presence of α-helical peptides. The peptide fraction was obtained through methanol, acetic acid and water mixture (90:1:9) extraction, followed by peptide purification using Reverse Phase High Performance Liquid Chromatography. The fraction exhibiting antibacterial properties was collected and characterized by Matrix-Assisted Laser Desorption/Ionization-Time of Flight. A linear α-helical peptide with flexible termini (LLKELWTKMKGAGKAVLGKIKGLL) was found, corresponding to a previously described peptide from ant venom and here denominated as *Bm*-ponericin-L1. The antibacterial activity of *Bm*-ponericin-L1 was determined against ESKAPE pathogens. Scanning electron microscopy confirmed the membrane disruption potential of *Bm*-ponericin-L1. Moreover, this peptide also showed promising antibiofilm activity. Finally, cell viability and hemolytic assays revealed that *Bm*-ponericin-L1 is non-toxic toward primary fibroblasts cell lines and red blood cells, respectively. This study opens up new perspectives toward an alternative approach to overcoming multiple-antibiotic-resistance by means of AMPs through invertebrates’ infection with human pathogenic bacteria.

## Introduction

**Figure 1:**
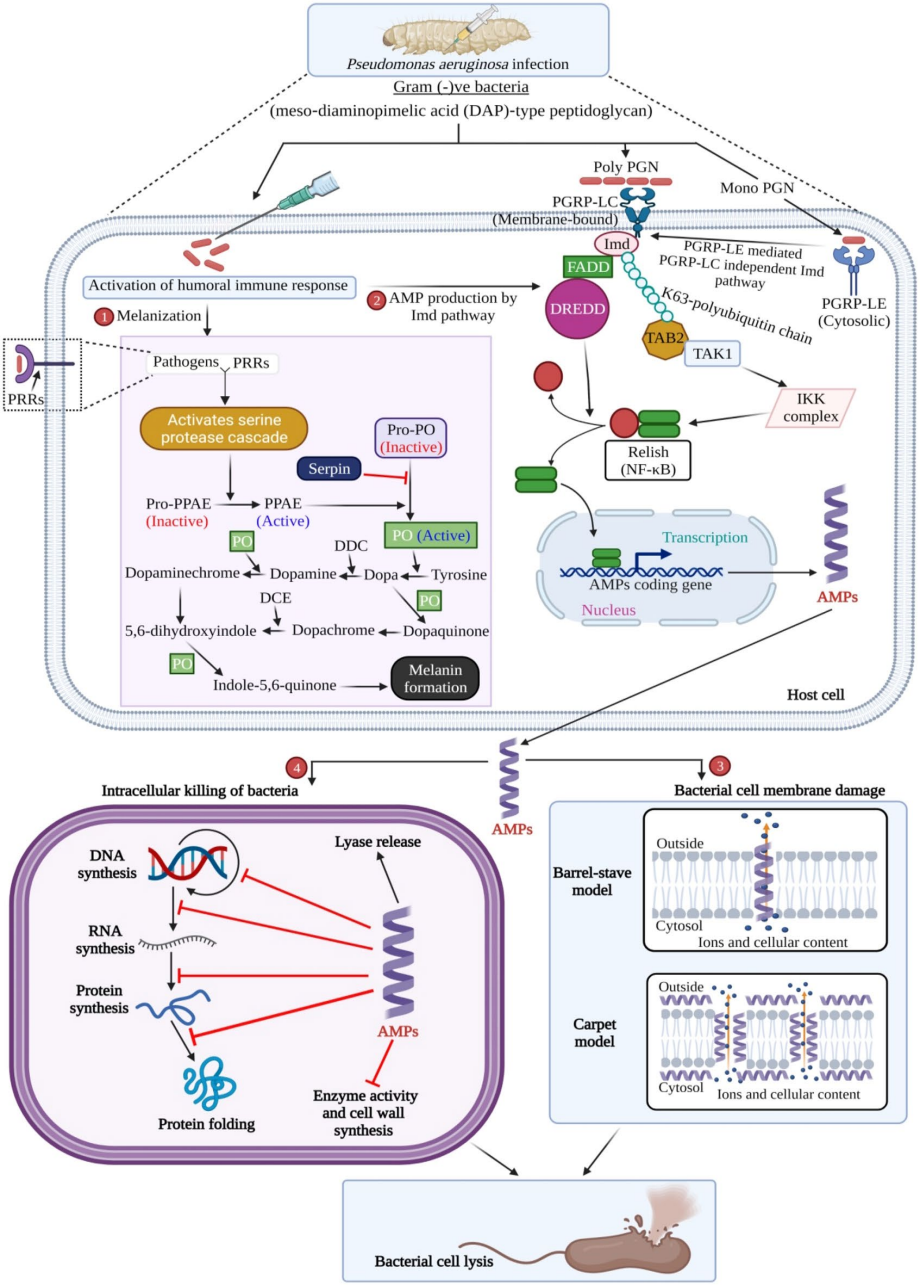
Silkworm immune response against Gram-negative bacteria through α-helical cationic AMPs. In *B. mori* L., Gram-negative bacteria or meso-diaminopimelic acid (DAP)-type peptidoglycan recognized by both pattern recognition receptors (PRRs), peptidoglycan recognition protein (PGRP)-LC (membrane-associated) and peptidoglycan recognition protein (PGRP)-LE (cytosolic) triggers sequential activation of melanization (1) and AMP production by IMD pathway (2), respectively. Melanin formation starts with the activation of the serine-protease cascade, which leads to the activation of the prophenoloxidase-activating enzyme (PPAE). The prophenoloxidase-activating enzyme (PPAE) helps to activate the phenoloxidase (PO) from prophenoloxidase (Pro-PO) (inactive). PO is actively involved in the melanization process through the formation of intermediate quinone products with the help of dopa decarboxylase (DDC) and dopachrome conversion enzyme (DCE). Serpin (serine protease inhibitor) inhibits PO formation. By contrast, the Imd pathway is also involved in AMP production upon Gram-negative bacteria infection. Polymeric DAP‐type peptidoglycan (Poly-PGN) binds to PGRP-LC protein (dimer), and monomeric DAP‐type peptidoglycan (Mono-PGN) binds to PGRP-LE protein. Extra and intracellular PGRP-LE activate the PGRP-LC dependent and independent Imd pathway, respectively. Imd interacts with FADD (Fas-associated protein with death domain) that, subsequently, recruits the caspase DREDD (FADD-death-related ced-3/Nedd2-like protein). DREDD cleaves Imd, which is then activated by K63-ubiquitination. The K63-polyubiquitin chains connect with TAB2 (TAK1-associated binding protein (2) to recruit and activate the TAK1 (transforming growth factor-beta (TGF-β)-activated kinase (1).TAK1 subsequently activates the IKK (inhibitor of B kinase) complex, which phosphorylates the NF-κB-like nuclear factor Relish. Free Relish moves into the nucleus and stimulates the transcriptionof AMP coding genes. AMPs produced during immunization with *P. aeruginosa* could cause bacterial cell death through two possible mechanisms, *i.e.*, bacterial cell membrane damage (3) and intracellular killing (4) by interaction with intracellular targets to compromise cell viability (Created with BioRender.com).

Insects' innate immune response involves cellular and humoral machinery localized in hemolymph to fight invading foreign pathogens (Fig. 1)^1^. For instance, insect hemocytes-mediated cellular responses are accountable for phagocytosis of the invading pathogens, nodulation, and encapsulation^2^. Moreover, the humoral immune response comprises melanization, reactive oxygen and nitrogen species production, and antimicrobial peptides (AMPs) expression (Fig. 1)^1^.

AMPs play a key role in insects' innate immune systems as they constitute the first line of defense against a diverse group of pathogenic microorganisms, including bacteria, fungi, and viruses^3^. Based on the amino acid composition and structural profiles, AMPs have been classified into several subgroups^4^. Some AMPs adopt an α-helical structural profile (α family), whereas others present β-sheet conformation (β family). AMPs can also present both α-helix and β-sheet in single structural scaffold (αβ family). Moreover, the absence of α-helix or β-sheet structures has also been reported for AMPs, comprising the non-αβ family (random coil peptides).

It is known that the invading fungi and bacteria accelerate systemic antimicrobial molecules production (*e.g.*, AMPs) through different protein cascades^5^. For instance, in the Toll pathway, the β-1,3-glucan from fungi and lysine-type peptidoglycans (Lys-PG) from Gram-positive bacteria activate serine protease cascade to enhance AMP production^6,7^. Diaminopimelic acid-PG (DAP-PG) from Gram-negative bacteria activates the immune deficiency (IMD) pathway through a protein complex (FADD, Dredd, and Relish) that transports NF-kB (a transcriptional factor) to the nucleus, accelerating the expression of AMP genes^1,3,8^. As a result, low-molecular-weight AMPs expressed during infections diffuse quickly through the target cell's plasma membrane and disrupt it without exerting toxic side effects towards the host’s cells.

Cationic AMPs bind to negatively charged bacterial cell membranes or cell wall, which may lead to pore formation that cause leakage of intracellular components and, ultimately, bacterial cell death^3^. AMPs are widely recognized as a potential class of antibiotics, especially for topical and surface applications. These molecules are also reported for adjuvant properties via restoring the antibacterial activity of antibiotics that have no longer been effective due to the emergence of bacterial resistance phenomena^9^. Currently, biofilm forming bacteria affects largely towards life-threating infections. AMP administration has proved to be effective against biofilm formation by its’ bactericidal activity or supressig virulence by inhibiting quorum sensing^10^. Therefore, AMPs represent a promising alternative to conventional antibiotics in developing a new strategy to overcome multidrug-resistant (MDR) pathogens.

Bearing this in mind, this study reports the isolation, characterization, and cytocompatibility of a low-molecular-weight peptide fraction present in *Bombyx mori* L. hemolymph upon *Pseudomonas aeruginosa* infection. To the best of our knowledge this is the first report of a ponericin-like peptide expressed and isolated from *B. mori.*

## Material and methods

### Silkworm rearing

*B. mori* eggs (Nistari, pure line) were collected from Hemtabad Sericulture farm (Ministry of Textile, Govt. of W.B, India). All the eggs were hatched, and larvae were reared in the Department of Sericulture, Raiganj University, India, maintaining 27 ± 1 °C temperature with 80 ± 10% humidity in 12 h light and 12 h dark conditions, daily. All the larvae were fed on S1635 mulberry (*Morus alba* L.) leaves to reach the 5th instar stage^11^.

### Bacterial inoculum preparation

*P. aeruginosa* (ATCC 25668) cells were stored at − 70 °C in 20% glycerol and cultured in nutrient broth (HiMedia; M002) at 37 °C for 18 h, for active culturing purposes.

### Physio-morpho-anatomical responesin *B. mori* induced by bacterial infection

One-hundred silkworm larvae (2–3 days old, 5th instar stage) were infected with 25 µL of *P. aeruginosa* (ATCC 25,668) cells at different bacterial loads (0.375 × 10^8^ CFU mL^−1^, 0.75 × 10^8^ CFU mL^−1^, and 1.5 × 10^8^ CFU mL^−1^) at the base of the first abdominal leg. Prior to bacterial infection, the inoculum size was determined following McFarland Standards.Twenty five larvae were used in each experimental group. As a control, 25 µL of phosphate buffered saline (PBS) (instead of the bacterial inoculum) was injected in twenty five silkworm larvae. Infected *B. mori* were collected and dissected at days one, two, three and four of incubation to examine the morphological, physiological, and anatomical changes in the digestive system and silk glands upon *P. aeruginosa* infection. Infected batches of *B. mori* were compared with non-infected batches kept as a control group. Ten silkworms were dissected for each experimental batch, and the average data was recorded. Further, to monitor the onset of melanisation after bacterial infection, the feces from the infected and non-infected *B. mori* were collected at 24 h and the polyphenol oxidase (PPO) activity was measured^12,13^. In this study, the commercially available *Drosophila melanogaster* prophenol oxidase 1 (DmPPO1) (1.25 µg) was used as a standard, for comparison. 200 µL aliquot of 10 mmol L^−1^ dopamine (Sigma) was used as substrate, whereas saturated phenyl thiourea (PTU) was used as inhibitor of PPO and DmPPO1. Before use, DmPPO1 was activated with ethanol (30%). Subsequently, 240 µg (wet weight) of feces were mixed with 200 µL of Tris buffer (10 mmol L^−1^, pH 7.4) and vortexed. 20 µL of these suspensions were thoroughly mixed with dopamine + saturated PTU and dopamine + Tris-buffer, thus adjusting the volume for each suspension. The dopamine with saturated PTU was used as blank. All the mixed solutions were incubated for 8 min at room temperature and centrifuged (10,000 × g for 1 min) to collect the supernatant. The absorbance of the supernatant was recorded at 490 nm, and the enzymatic activity was measured as ΔAλ/min = 0.001 (λ = wavelength).

### Hemolymph collection

At 4 h, 16 h and 24 h intervals, hemolymph was collected in a pre-chilled microtube containing a pinch of PTU by rupturing the first abdominal leg with a sterile needle. Immediately, phenylmethane sulfonyl fluoride (PMSF; 1 mmol L^−1^) was added to the hemolymph to prevent peptide degradation. The collected infected and non-infected hemolymyph was centrifuged (3000 × g for 10 min, at 4 °C) to remove hemocytes. The supernatant was collected and stored at − 40 °C for further investigation. The hemolymph was tested for antibacterial activity against *P. aeruginosa* (ATCC 25668) by agar well diffusion assay for inoculum size optimization, which triggers AMP production. As a control set, non-infected hemolymph was used for zone of inhibition test to compare our results. The pure culture of *P. aeruginosa* (ATCC 25668) was sub-cultured in Luria–Bertani (LB) broth. Wells were prepared on LB agar plates using gel puncture and poured with purified hemolymph. After 24 h of incubation, the zone of inhibition was measured. Three independent experiments were performed for each condition tested.

### Partial purification of peptides

We used the methods reported by Schoofs et al. (1990) to prepare an acidic methanolic extract from *B. mori* hemolymph^14^. Briefly, hemocytes-free plasma was diluted ten times in methanol-acetic acid (MGW) extract [methanol (90): glacial acetic acid (1): distilled water (9)], mixed, and kept at room temperature for 10 min and centrifuged at 10,000 × g for at least 30 min at 4 °C. The supernatant was collected, and the methanol was evaporated using a rotary evaporator. The remaining solution was lyophilized and dissolved in 0.1% trifluoroacetic acid (TFA). To remove the lipid content, the same volume of n-hexane was added, mixed thoroughly, and centrifuged at 10,000 × g for 10 min at 4 °C. The upper fraction containing the lipid was discarded, and the lower polar fraction containing peptides were collected, freeze-dried, and further dissolved in 0.1% TFA.

### UV–vis and FTIR study

Initially, the spectroscopic study of MGW extracts of hemolymph was done by UV–visible spectrophotometer (Varian Inc., USA) within the range of 190–900 nm using quartz cuvette of 1 cm of optical path length, as described previously^15^. FT-IR analysis of the MGW extracted hemolymph fractions were analysed using FTIR spectrophotometer (Thermo Scientific Nicolet 380) equipped with a Helium–Neon laser, deuterated triglycine sulphate detector, and a KBr beam splitter in the wavelength range of 4000–500 cm^−1^, at room temperature. A small amount of hemolymph was taken in the glass capillary and added to the dry KBr powder, and then a pellet was prepared and scanned to get FTIR spectrum^15^.

### RP-HPLC study of MGW extracted hemolymph

The MGW extracted hemolymph was examined via reverse-phase high-pressure liquid chromatography (RP-HPLC; Waters TM HPLC system) with a C-18 Prep column. Two solvent sets (Solvent A & Solvent B) were used, solvent A: 0.1% TFA in water (v/v) and solvent B: 0.1% TFA in 100% acetonitrile (v/v). A linear gradient of 0–100% acetonitrile was used to collect different fractions over 30 min, flow rate of 8 mL min^−1^, monitored at 254 nm. All the fractions were collected and freeze-dried to perform antibacterial assay. The active fraction showing antimicrobial activity was further purified through C-18 Prep column as described above, collected and stored at -80 °C for further characterization.

### Mass spectrometry and peptide identity

The lyophilized AMP was re-suspended in 0.1% TFA. 2 μL of the peptide solution was mixed with 2 μL of 4-HCCA matrix (10 mgmL^−1^), and spotted onto the sample plate, dried and analyzed^16^. To obtain MALDI mass spectra, a MALDI-TOFmass spectrometer with Bruker Daltonics GmbH autoflex speed (Bruker, Germany) operated in accelerating voltage 20 kV was used. The spectra were recorded in positive ion linear mode with mass range of 600–3400 Da. Reproducibility of the spectrum was checked numerous times from separately spotted samples. Further, the data was uploaded into MASCOT (Matrix Science, London, UK) database to search the peptide identity. Before baseline correction, the spectra were normalized with dividing each spectrum by its mean value. Secondly, the spectra were subjected to baseline correction with a correlation factor of 0.7 and Gaussian smoothing to reduce noise using 5-point filter width. Finally, the search parameters were defined as: database, SwissProt; no cleavage by enzyme; allowing no missed cleavage; peptide mass maximum variation of 0.5 Da.

### Purified peptide in silico characterization

The peptide sequence identified by MALDI was submitted to computational analyzes to characterize its physicochemical properties, predict antibacterial activities, and generate three-dimensional theoretical models. The net charge, hydrophobicity, hydrophobic moment, and helical wheel diagram were generated using the HeliQuest server (https://heliquest.ipmc.cnrs.fr)^17^. For antibacterial activity prediction, Support Vector Machine (SVM), Random Forest (RF), Artificial Neural Network (ANN) and Discriminant Analysis (DA) algorithms from CAMP_R3_ were used^18^. Additional tools were also selected for antibacterial prediction, including the DBAASP server, as well as a recent predictor called Sense the Moment (STM)^19,20^.These analyzes were also performed for all ponericin-like peptides deposited in the Antimicrobial Peptide Database (APD), for comparison.

### Molecular modeling and structural refinement

The atomic coordinates for the purified peptide were obtained using the AlphaFold2 server^21^. The lowest free-energy theoretical model had its structural statistics obtained through the ProSA-web server, PROCHECK and MolProbity^22,23^.These same analyzes were also performed for the 16 ponericin-like peptides retrieved from APD, aiming at classifing our lead peptide according with its structure.The final structure was then refined through unconstrained molecular dynamics (MD) simulations in saline solution (ionic strength 0.15 mol L^−1^ NaCl). The simulations were performed using the GROMOS96 43A1 force field from the computer package GROMACS v.5.0.4^24^. The geometry of the water molecules was constrained using the SETTLE algorithm; whereas the LINCS algorithm was used to bind all atomic binding lengths^25^. Particle Mesh Ewald (PME) was used for electrostatic corrections, with a cutting radius of 1.4 nm to minimize the computational simulation time. The same cutting radius was also used for the van der Waals interactions. The neighbor list of each atom was updated every 10 simulation steps of 2 fs each, the system was subjected to 50,000 energy minimization steps using the steepest descent algorithm. The systems underwent two normalizations of temperature (298 K and 310 K) and pressure (1 bar) using the speed rescheduling thermostat (NVT set) and the Parrinello-Rahman barostat (NPT set), respectively, to 100 ps. Systems with minimized energy and balanced temperature and pressure were subjected to structural refinement trough 40 ns using the leapfrog algorithm as an integrator. The resulting structures at different temperatures were visualized using PyMOL^26^.

The solvation potential energy calculation was measured for the lowest energy three-dimensional theoretical structures generated by molecular modeling and structure refinement. The conversion of .pdb files into .pqr files was performed on the PDB2PQR server using the AMBER force field^27^. The grid dimensions for Adaptive Poisson-Boltzmannsolver (APBS) calculation were also determined by PDB2PQR. Solvation potential energy was calculated on APBS, with potentials ranging from − 5 kT/e to + 5 kT/e. Surface visualization was performed using the APBS plugin for PyMOL^26^.

### Antimicrobial and antibiofilm activity

*Enterococcus faecium* (ATCC 35667)*, Staphylococcus aureus* (ATCC 6538)*, Klebsiella pneumoniae* (ATCC 70063)*, Acinetobacter baumannii* (ATCC 17978), *P. aeruginosa* (ATCC 10145), and *Enterobacter agglomerans* (ATCC 27985) belong to the ESKAPE bacterial group and were cultured in Mueller Hinton Broth (MHB) to determine the minimum inhibitory concentration (MIC) of the *Bm*-ponericin-L1 peptide using broth microdilution technique as per Clinical and Laboratory Standards Institute (CLSI) guidelines^28^. Cell suspensions of each ESKAPE pathogens were adjusted to attain the required cells mL^−1^ by measuring the turbidity of cell suspensions using spectrophotometer (Multimode Reader Synergy Neo 2, Biotek, USA). The antimicrobial assay was performed in 96-well microtiter plate using different concentrartion of *Bm*-ponericin-L1 to determine 50% inhibition of microbial growth (IC50). The turbidity of each bacterial suspension was adjusted to an OD = 0.003 at 600 nm (∼100-fold dilution of parent culture) to obtained standardized inoculum. 190 μL of the test-adjusted cell suspension of the tested organism and 10 μL of different concentrations (parts per million—ppm) of *Bm*-ponericin-L1 were added to a microtiter plate. MHB containing only bacterial inoculum and MHB containing bacterial inoculum along with gentamicin were used as negative and positive control, respectively. The plates were incubated at 37 °C and the MICs of *Bm*-ponericin-L1were expressed as an IC_50_ value. All the experiments were carried out in triplicate and the mean ± standard deviation was calculated. The percentage of bacterial growth inhibition by varied doses of *Bm*-ponericin-L1 was calculated using equation [(Ac-At)/Ac] × 100, where Ac and At were the absorbance at 600 nm of negative control and treated samples, respectively. In addition, the strain-dependent antimicrobial effect of *Bm*-ponericin-L1 peptide was also checked against *P. aeruginosa* (ATCC 25,668). MHB containing bacterial inoculums only was used as negative control, whereas MHB contining bacterial inoculums along with gentamicin was used as postive control. To determine the changes in morphology of *P. aeruginosa* (ATCC 10145) after *Bm*-ponericin-L1 peptide treatment, scanning electron microscpy (SEM) analysis was performed as previously described, with modifications^29^. Briefly, overnight cultures of *P. aeruginosa* (ATCC 10,145) was washed and suspended in PBS, incubated with *Bm*-ponericin-L1 at 37 °C, for 6 h. Following incubation, bacterial cells were fixed with 2.5% glutaraldehyde and dehydrated using a series of ethanol treatments and examined by Hitachi S-3400 with accelerating voltage of 20.0 kV. Multiple fields of visions were observed at different magnifications.

The antibiofilm activity of the *Bm*-ponericin-L1 peptide was calculated following the mehods described previously^29^. Biofilm inhibition (in %) was calculated using following equation:

$$ {\text{Biofilm inhibition }}\left( {{\text{in }}\% } \right) \, = \, \left[ {{1} - \left( {{\text{OD}}_{{{62}0}} {\text{of cells treated with ponericin}} - {\text{L1}}/{\text{OD}}_{{{62}0}} {\text{of non}} - {\text{treated control}}} \right) \, \times {1}00} \right]. $$

### Hemolytic assay

Hemocompatibility of the purified peptidewas evaluated through hemolytic assay, as described previously^30^. The diluted bovine red blood cells (RBCs) (0.2 mL) were mixed with the purified peptide at 2 ppm and incubated at ambient temperature for 2 h. The % hemolysis was calculated by measuring the absorbance of the supernatant at 541 nm in an UV−vis spectrophotometer (Varian Inc., USA), and using the following equation:

$$ \% {\text{ hemolysis }} = \, \left( {{\text{A}}_{{\text{S}}} - {\text{A}}_{{\text{N}}} } \right)/\left( {{\text{A}}_{{\text{P}}} - {\text{A}}_{{\text{N}}} } \right) \, \times { 1}00. $$

where, A_S_, A_N_, and A_P_ are the absorbance of the sample, negative control, and positive control, respectively.

### Cytocompatibility assay

The primary fibroblasts cell lines (ATCC PCS-201-012) were incubated in a 96-well microplate with DMEM/F-12 media and 5% CO_2_ at 37 °C, for 24 h. To evaluate the cytotoxic effect of *Bm*-ponericin-L1 (from 0.25 to 8 ppm), 10^5^ cells per well were seeded in a 96-well plate. After the cellular confluency was completed, the serially diluted peptide solutions were applied on the surface and incubated for 24 h at 37 °C. Thereafter, freshly prepared 3-(4,5-Dimethylthiazol-2-yl)-2,5-Diphenyltetrazolium Bromide (MTT) reagent was applied to each well and incubated for 3 h. During incubation, the MTT reagent enters the mitochondria of the fibroblasts cells and it is transformed into formazan (insoluble) by dehydrogenase. Then, the formazan was solubilised in isopropanol and the absorbance was recorded at 570 nm using a Biotek Neo 2 System microplate reader. The tissue culture polystyrene (TCPS) was used as a control group.

### Statistical analysis

The data were analysed using one-way ANOVA and the values were expressed as mean ± standard deviation. The significance level was set at < 0.05.

## Results and discussion

### Effects of inoculum size on AMP production

The hemocyte-free hemolymph was checked for antibacterial activityon LB agar plates seeded with *P. aeruginosa* (ATCC 25668). All the hemolymph samples were collected at 4 h, 16 h, and 24 h from different batches of infected and non-infected *B. mori*. As a result, all the control group samples did not exhibit any antibacterial activity. Moreover, hemolymph collected after 4 h or 16 h of infection did not exhibit any zone of inhibition. By contrast, hemolymph collected after 24 h of infection showed zone of inhibition suggesting it’s inoculum-dependent and time-dependet activity, which may be correlated with AMPs production. Additionaly, we observed that all the three bacterial loads (0.375 × 10^8^ CFU mL^−1^ to 1.5 × 10^8^ CFU mL^−1^) triggered AMP production with different inhibition zones (Table 1). It was observed that hemolymph isolated from the silkworm infected with 1.5 × 10^8^ CFU mL^−1^ for 24 h exhibited the highest antibacterial activity against the tested bacterium. Thus, this infected group was selected for AMP purification and characterization.

**Table 1:** Effects of incubation time and inoculum size on AMP production by *B. mori* L., and its antibacterial properties against *P.aeruginosa* (ATCC 25668).

Incubation time (h)	Inoculum size (CFU mL^−1^)	Inhibition zone (cm)	LB plates showing inhibition zones	Positive control (Norfloxacin- NX)
4	0	–		
	0.375 × 10^8^	–		
	0.75 × 10^8^	–		
	1.5 × 10^8^	–		
16	0	–		
	0.375 × 10^8^	–		
	0.75 × 10^8^	–		
	1.5 × 10^8^	–		
24	0	–		
	0.375 × 10^8^	1.20		
	0.75 × 10^8^	1.30		
	1.5 × 10^8^	1.53		Zone of inhibition: 2.1 cm

### Changes in physio-morpho-anatomical responses in *B. mori* during bacterial infection

**Figure 2:**
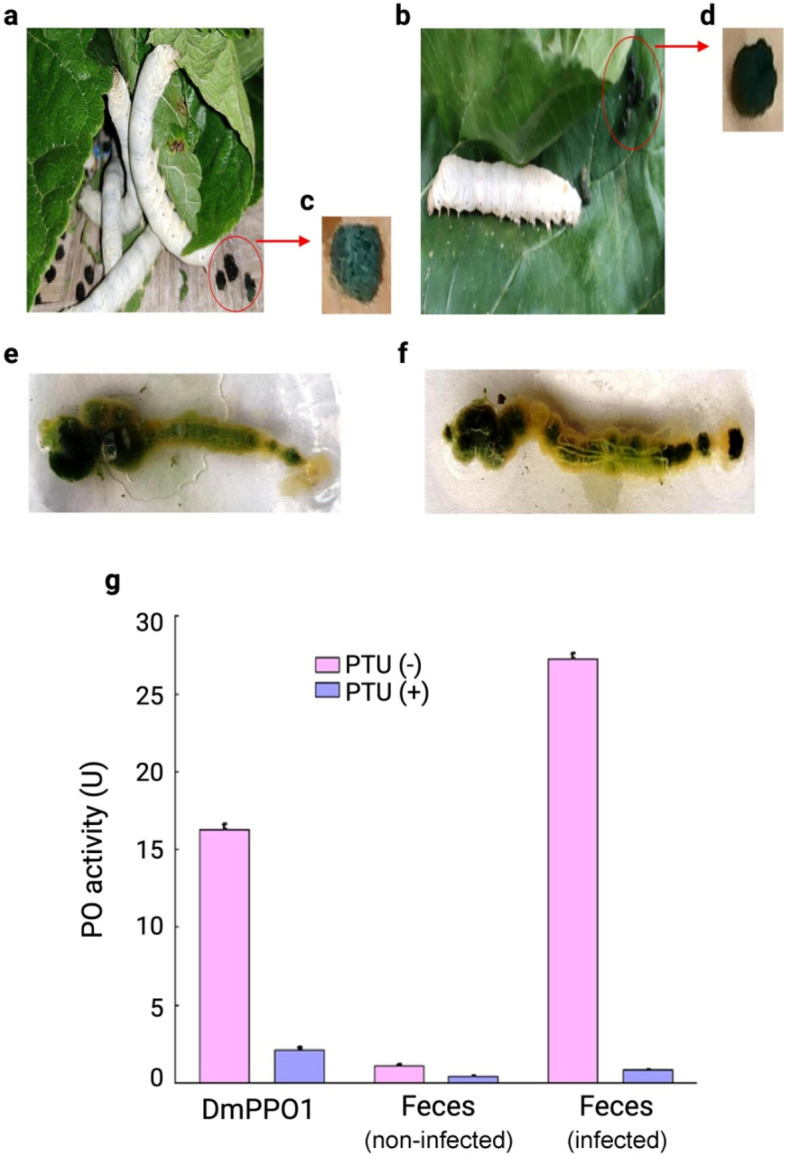
Fecal melanization in non-infected and infected *B. mori* larvae. (**a**) Non-infected larvae with continued feeding, (**b**) infected larvae that stoppted eating, (**c**) green-colored feces of non-infected larvae, (**d**) black-colored feces of infected larvae, (**e**) dissection showed green gut content in the non-infected larval group, (**f**) black gut content in the infected larval group, and (**g**) polyphenol oxidase (PPO) activity of non-infected and infected silkworm feces. The data were expressed as mean ± standard deviation and the significance level was set at *p* < 0.05.

The present investigation revealed that non-infected group silkworm larvae fed on S1635 mulberry leaves excreted green-colored feces, whereas the infected group excreted black-colored feces (Fig. 2a–d). After dissecting the third day of fifth instar infected or non-infected larvae (24 h), we observed that the alimentary canal content of non-infected silkworm was green (Fig. 2e). By contrast, the alimentary canal content of infected silkworm was found to be well-darkened towards the cephalo-caudal end (Fig. 2f). Melanization immune response is a principal element of insect immune defense against invading pathogens^13,31,32^. Following the microbial invasion, PPO is converted into active phenoloxidase (PO), a process mediated by serine proteases (SPs)^33–36^. PO participates in foreign pathogens elimination by Toll pathway activation^34^. PO is a critical enzyme in *B. mori* that induces melanization cascades at wounds or around the invading pathogen^37^. Melanization is correlated with high PO activity in hindgut content and the darkening of feces. Here, the enzymatic assay revealed that, when dopamine (phenoloxidase substrate) was incubated with *D. melanogaster* DmPPO1 in the absence of PTU (PPO inhibitor), high oxidative enzyme activity by DmPPO1 is observed (Fig. 2g). However, in the presence of an inhibitor, DmPPO1 loses its activity to a significantly low level. Green-colored feces from non-infected silkworm larvae did not show any significant enzymatic activity, confirming the absence of PPO (Fig. 2g).

Black-colored feces from infected larvae exhibited very high phenoloxidase activity (*p* < 0.001) that, in turn, oxidized dopamine. This enzymatic activity was significantly lost in the presence of PTU validate the presence of PPO in the gut content and wet feces of infected larvae, resulting in the melanization and, ultimately, blackening of feces. Change into the green to black color of feces is also due to dehydration of hindgut content, which may be attributed due to the polymerization of hydrophobic polyphenols into the feces. Along with the toxic properties of oxidized phenol, the dehydration of gut content might be responsible for decreasing the pathogen's load^13^. It is known that melanization in infected silkworm confirms the innate immune response due to bacterial invasion, which gradually activates protein kinase cascade through Toll and IMD pathways to accelerate AMP production (Fig. 1)^1,3,38^.

Our study showed that silkworm larvae challenged with *P. aeruginosa* also induced modification in physiological processes, leading to significant morpho-anatomical changes. It was noted that infected silkworms immediately stopped feeding, which may be due to mechanical shock, whereas the non-infected silkworm showed normal foraging behavior (Supplementary Fig. S1a–b). It was also observed that the infected larvae showed swelling in the head and thorax region (Supplementary Fig. S1c). On the second day of infection, there were changes in the integument color from white to pale yellow (Supplementary Fig. S1d). On the third day of infection, the integument turned into coffee-brown color (Supplementary Fig. S1e) with minimal foraging behavior. During the fourth and fifth days, the larval outer integument turned black, ultimately ruptured, and died due to hemolymph leakage (Supplementary Fig. S1f).

**Figure 3:**
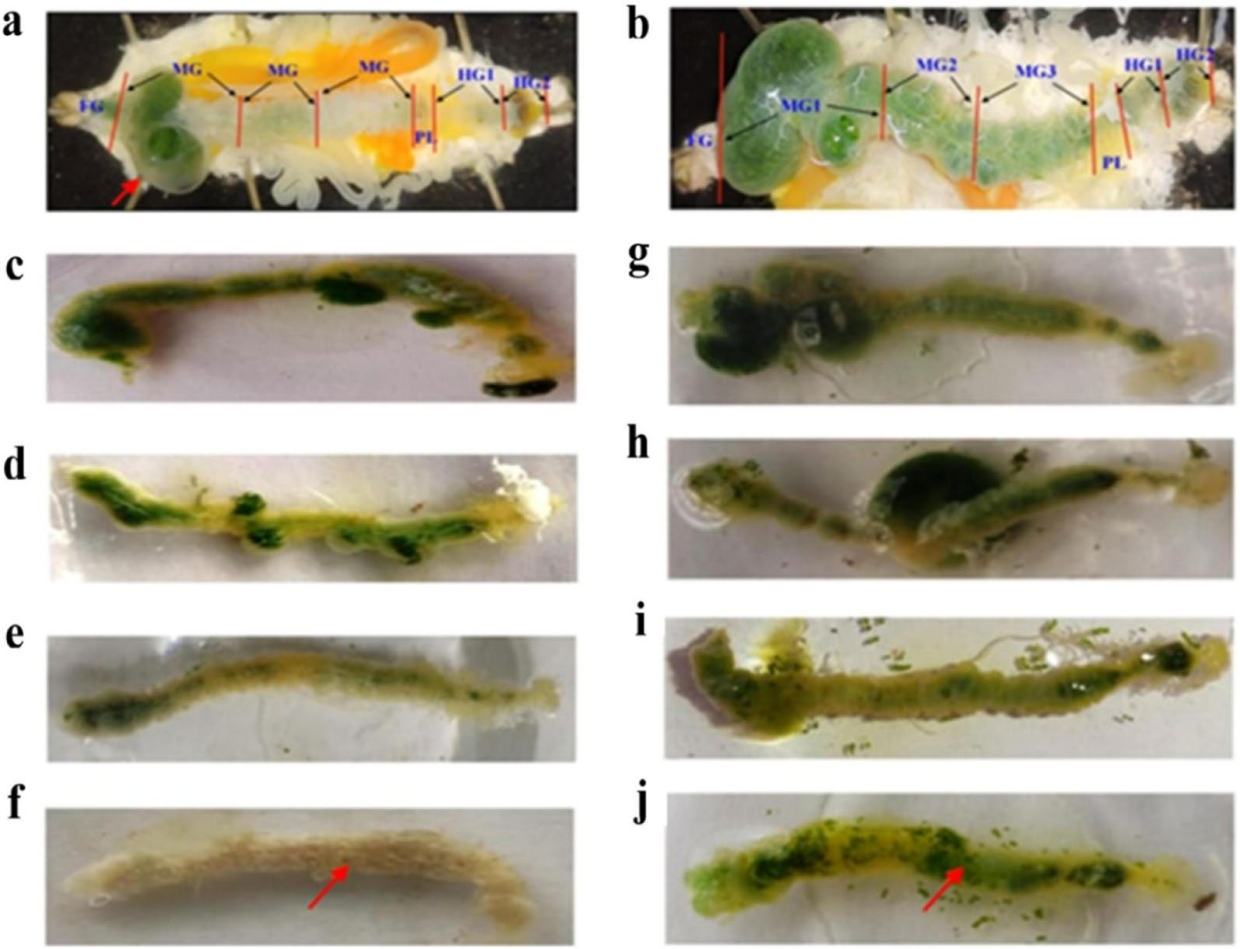
Anatomical comparison between infected and non-infected *B. mori* larvae. (**a**) Red arrow showing gut content accumulation in the infected larvae. (**b**) Control larvae showed green gut content throughout the alimentary canal (FG, MG1, MG2, MG3, PL, HG1, and HG2). (**c**–**f**) The green color of gut content in the alimentary canal decreased gradually and appeared complete white in fourth day of infected larvae. (**g**–**j**) Control larvae showed no changes in gut color.

Our comparative anatomical study revealed that, on the second day of infection, there was an accumulation of food content in the foreguts (FG) and the first part of the midgut (MG1) with traces of melanized feces in the hindgut (HG). As a comparison, the non-infected silkworms were also dissected (Fig. 3a,b). We further recorded that the gut content gradually turned from green to white due to the modulation in foraging behavior and appetite loss in infected silkworms from the first to the fourth day of infection (Fig. 3c–f). Control non-infected silkworms exhibited no gut color changes (Fig. 3g–j).

The appetite loss triggered by bacterial infection cause significant decrease in both wet gut weight and silk gland compared to the control group (Supplementary Fig. S2a–b). Our study also revealed that due to the modulation in foraging behavior in the treated group, there is a decrease in silk production resulting in white color silk gland (Supplementary Fig. S2c–f). By contrast, the silk gland of the dissected non-infected group showed a gradual increase in the intensity of yellow color, which is probably due to normal feeding behavior accompanied by increased silk production (Supplementary Fig. S2g–j).

As reported earlier, the pathogenic infection causes significant decrease in larval body weight, somatic index of silk gland tissue and weight of silk gland, which supports our findings^39,40^. A separate study on PPO activity in *B. mori* revealed that pathogenic infection leads to the immune induction via PPO activation cascade^41^. After pathogenic infection, hindgut cells of silkworm larvae produces PO, triggering blackening and dehydration of both hind gut content and feces^13^. Another study showed that knock-down and knock-out of PPO gene seriously alters its immune capacity and longevity in insects, thus indicating the significance of insect PPO in immune protection and it’s correlation with AMPs production^42^.

### UV–vis and FTIR study of MGW extracted hemolymph

**Figure 4:**
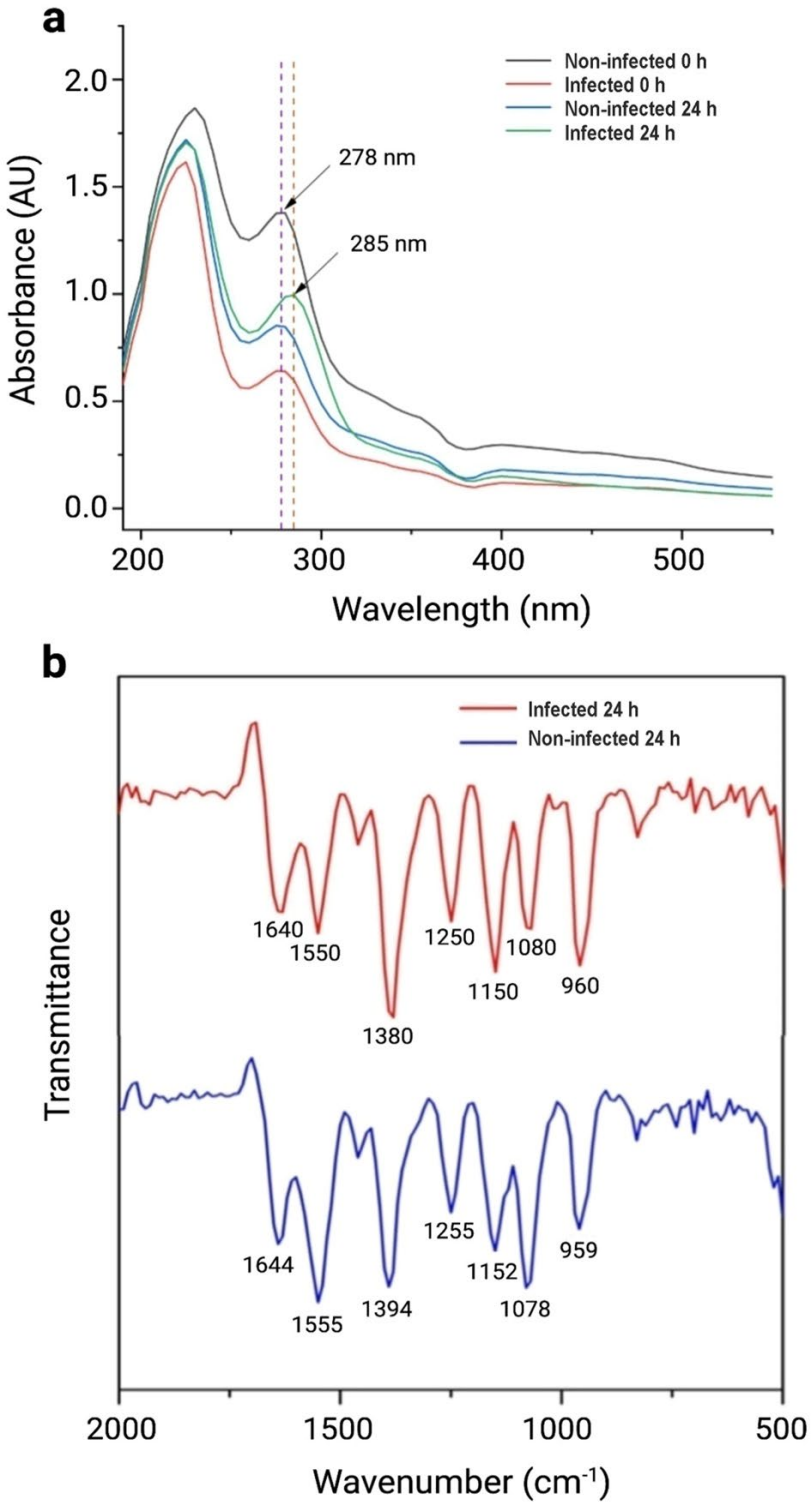
UV–vis spectra and FTIR analyses. (**a**) UV–vis spectra and (**b**) FTIR spectra of hemolymph from non-infected and infected silkworms.

The protein backbone comprises a peptide chain with aromatic amino acids, including tyrosine, tryptophan, and phenylalanine, which can absorb in the UV region because of the presence of π electron cloud. The absorbance of ultraviolet radiation at 280 nm indicates the presence of amino acids having aromatic rings and, sometimes, disulfide bonds to a small extent^43^. Moreover, the peaks around 220 nm correspond to protein absorption. UV–vis spectra of non-infected hemolymph and infected hemolymph of *B. mori* after 0 h and 24 h, respectively, was recorded. The UV absorption peak at 278 nm was observed for non-infected 0 h, infected 0 h, and non-infected 24 h. Nevertheless, a sharp change of λ_max_ from 278 to 285 nm (bathochromic shift) was observed for 24 h incubated infected silkworm hemolymph, which might be attributed to its chemical changes during bacterial infection (Fig. 4a).

FTIR spectroscopy is a widely used method to investigate the structure of proteins and peptides either isolated or in a complex sample (*e.g.*, insect’s hemolymph). The FTIR spectra of non-infected and infected hemolymph from *B. mori* showed characteristics peak of amide bonds at different regions. The peaks at 1644 cm^−1^ and 1640 cm^−1^ represent the characteristic peak of C = O stretching vibration of the amide I band of non-infected 24 h and infected 24 h hemolymph, respectively (Fig. 4b)^44^. Similarly, the peaks at 1555 cm^−1^ and 1550 cm^−1^ represent the amide II band of non-infected 24 h and infected 24 h hemolymph, respectively (Fig. 4b). Additionally, two peaks at 1255 and 1250 cm^−1^ indicate the presence of amide III absorptions, which are very weak in infrared spectra and arise from N–H bending^44^. A clear shift of the three characteristics regions of possible peptide chain of non-infected 24 h and infected 24 h hemolymph is attributed to some chemical changes during the infection. No peak was found at 1700–1750 cm^−1^, indicating the C-terminal amino acid in possible peptide chains is not esterified. The peak at 1550 cm^−1^ of infected 24 h strongly indicates the presence of α-helical peptides (with possible antibacterial properties) in the hemolymph. Finally, the absorption at relatively lower wave numbers suggests the presence of water in the hemolymph^45,46^.

### HPLC and Mass spectrometry

**Figure 5:**
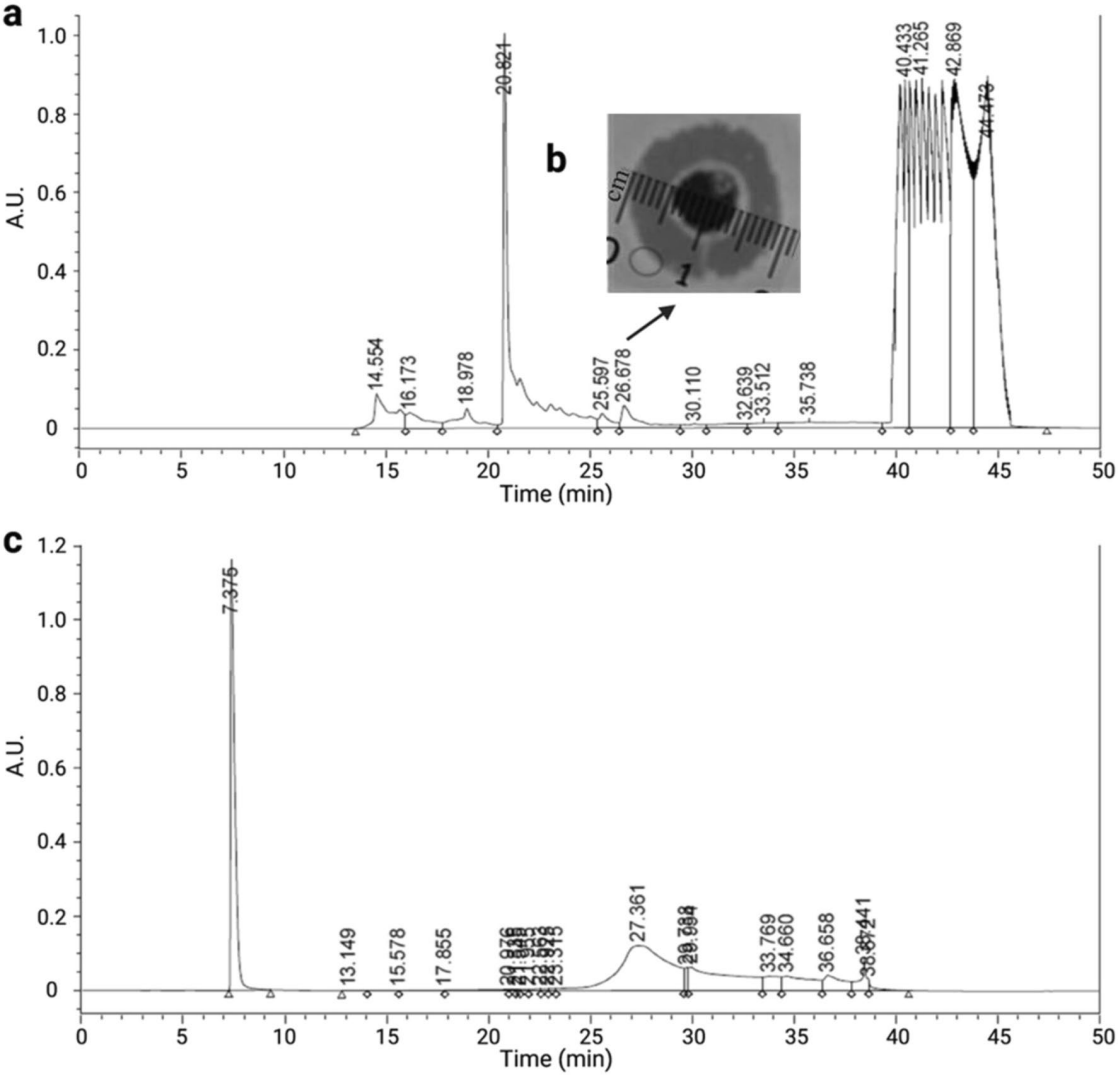
Purification of *B. mori* hemolymph infected with *P.aeruginosa*. (**a**) RP-HPLC fractionation of MGW extracted hemolymph through C-18 Semi-Prep column, (**b**) The arrow indicating the fraction exhibited antimicrobial activity (inhibition zone 1.9 cm), (**c**) RP-HPLC chromatogram of the active peak.

The MGW extracted hemolymph was purified through RP-HPLC for 50 min. The HPLC chromatogram (Fig. 5a) shows several peaks, where each one was collected and tested for antimicrobial activity against *P. aeruginosa* (ATCC 25,668). The fractions collected at 26.67 min (fraction 6) showed antimicrobial activity (inhibition zone of 1.9 cm) against the tested bacterium (Fig. 5b) and, therefore, was selected for a second round of purification through RP-HPLC (Fig. 5c). The MIC value of the isolated peptide was found to be 2 ppm against *P. aeruginosa* (ATCC 25,668). This MIC is in agreement with the results obtained from previous studies, where the antibacterial of numerous AMPs, including Q53, *Bm*Cec B, PA13, E53 *Bm*Cec B, Melimine and Mel4 were determined against *P. aeruginosa* at 2.2 ppm, 3.91 ppm, 4 ppm, 66 ppm and 106.5 ppm, respectively^47–49^.

**Figure 6:**
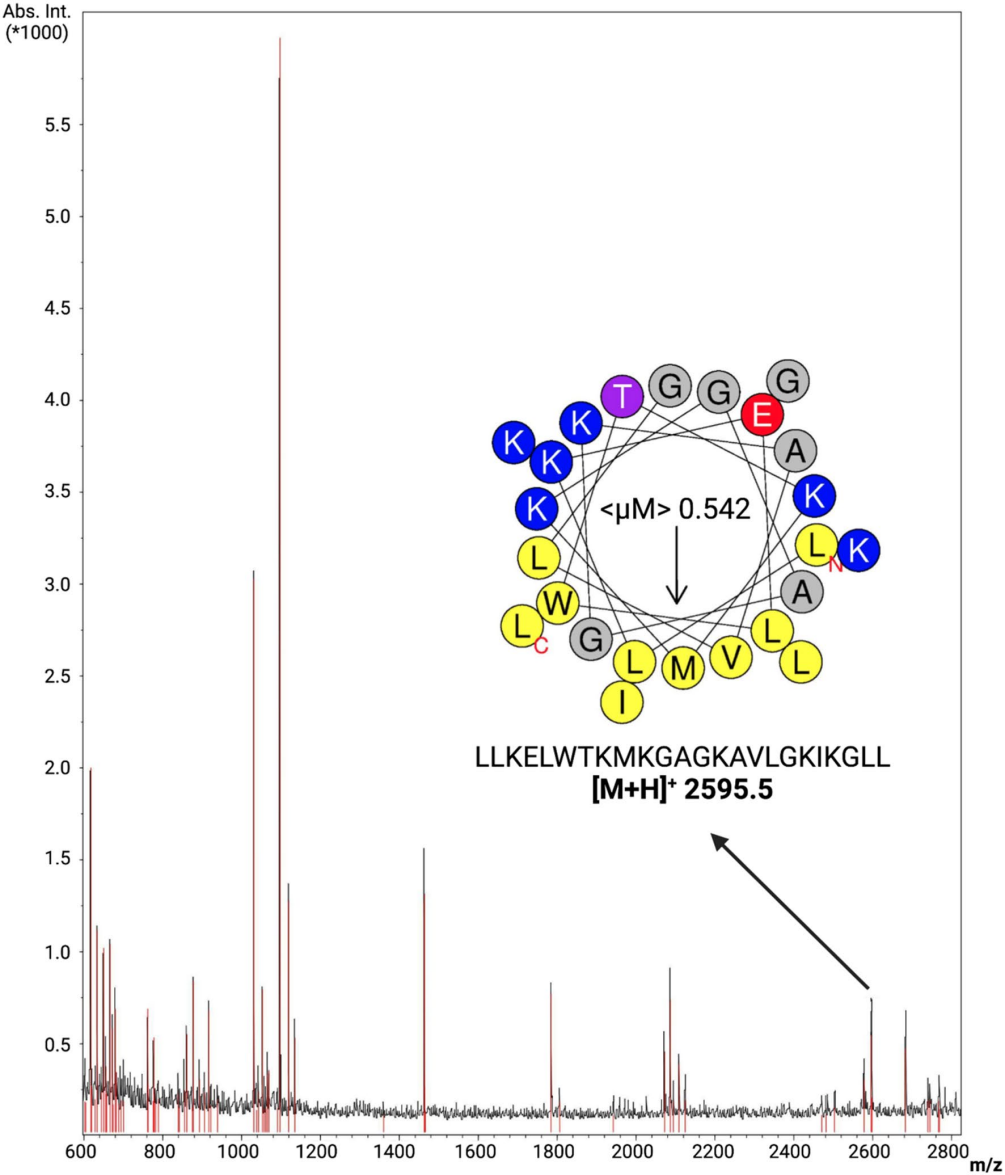
Characterization of *Bm*-ponericin-L1through MALDI-TOF. An ion of monoisotopic mass [M + H]^+^ of 2595.5 m/z is represented, corresponding to the peptide sequence LLKELWTKMKGAGKAVLGKIKGLL, previously described as ponericin L1 (an AMP isolated from ant venom). The helical wheel diagram is also shown. < μM >  = hydrophobic moment.

The fractions were collected from 25 to 29 min and analyzed via Matrix-Assisted Laser Desorption Ionization (MALDI), aiming at characterizing the purified peptide. Among the ions submitted to MASCOT analysis, a peptide with molecular mass MH + 2595.61 Da was obtained (Fig. 6). This ion corresponds to a 24-amino acid residues peptide (LLKELWTKMKGAGKAVLGKIKGLL), as revealed through MALDI-TOF followed by MASCOT analysis. Here, this peptide was initially named *Bm*-Frac6 (*B. mori* fraction 6) (Supplementary Table S1). This peptide sequence presented a complete match with an AMP deposited in APD, ponericin-L1 (APD ID: AP00383). This ponericin-like peptide was firstly isolated from ant venom and, due to its completely identity with *Bm*-Frac6 from this study, we renamed our lead peptide candidate *Bm*-ponericin-L1 (*B. mori* ponericin-L1).

### Purified peptide in silico characterization

The peptide sequence described above was submitted to a suite of computational analyzes. The *Bm*-ponericin-L1 peptide consists of a linear, cationic (net charge + 5) peptide, with a hydrophobic rate of 45.8% and a hydrophobic moment of 0.542 based on the Eisenberg scale, thus indicating amphipathicity. Apart from characterizing the physicochemical properties for *Bm*-ponericin-L1, we also retrived all the 16 ponericin-like peptides deposited in APD and submitted their sequences to those same calculations, as show in Supplementary Table S2. In general, the ponericin-like peptides present a net-positive charge ranging from 2 to 7, overall hydrophobicity from 22% to 60.8% (the latter is at the limit value acceptable for potential AMPs that do not compromise the viability of healthy mammalian cell lines), and hydrophobic moment varying from 0.228 to 0.587 (the higher the < μM > the greater the amphipathicity) (Supplementary Table S2). In terms of antibacterial properties, SVM, RF, ANN and DA algorithms from CAMP_R3_ predicted the *Bm*-ponericin-L1 peptide as a potential AMP (overall probability = 99.4%). These predictions were reinforced by the DBAASP prediction tool, as well as STM (prediction score = 88.1%). Our computational data is further strengthened by previous reports regarding the antimicrobial potential of the ant venom-derived ponericin-L1^50^. Additionally, those same algorithms were applied to the other ponericin-like sequences from APD, indicating that only ponericin-G4 and ponericin-W6 did not attend the criteria of ANN and DBAASP for antimicrobial activity, respectively. Nevertheless, it is worth noting that all the ponericin-like peptides were predicted as AMPs by most of the prediction algorithms used, except for ponericin-G4, which presented the lowest probabilities and scores (Supplemantery Table S3).

**Figure 7:**
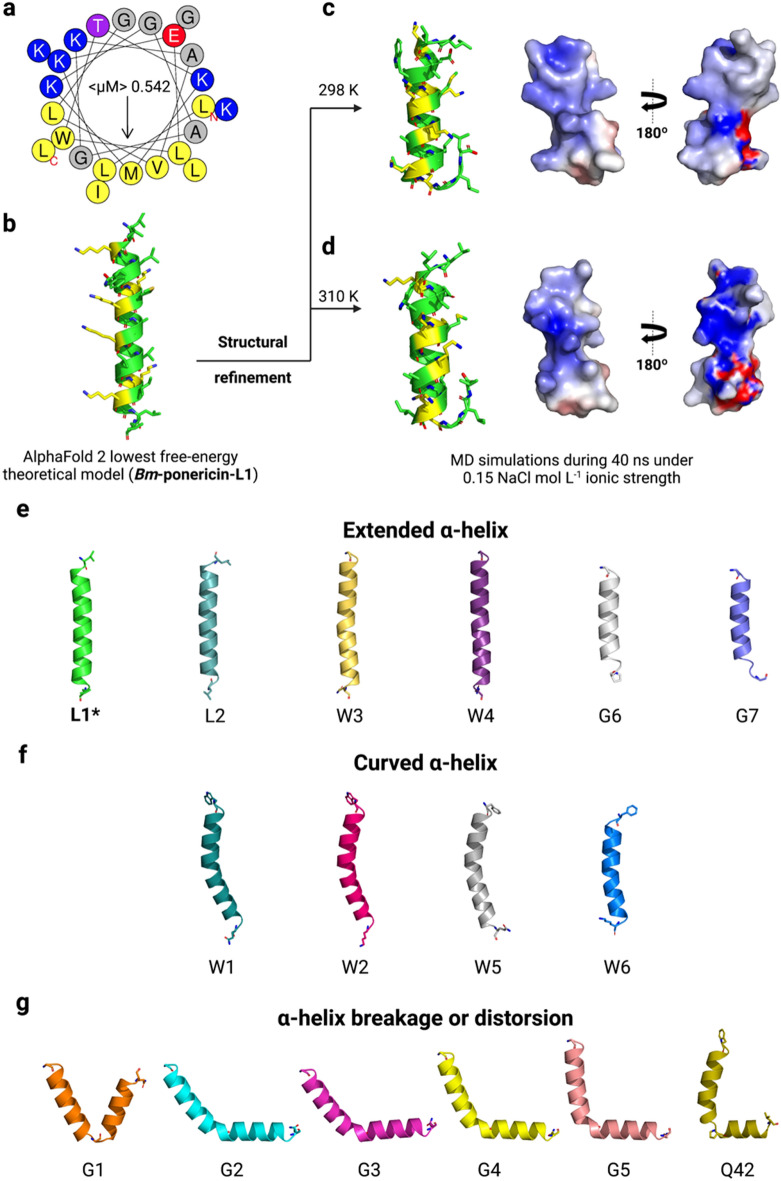
In silico structural characterization of *Bm*-ponericin-L1. (**a**) The helical wheel diagram for *Bm*-ponericin-L1is shown, along with its lowest free-energy three-dimensional theoretical model. (**b**) The structural refinement was performed at 298 (**c**) and 310 K (**d**), and 0.15 NaCl mol L^−1^ ionic strength through MD simulations (40 ns). A total of 16 ponericin-like peptides were retrieved from APD and submitted to molecular modeling. These peptides were divided into three main classes of structure profiles, including (**e**) extended α-helix, (**f**) curved α-helix and (**g**) α-helix breakage or distorsions.

Considering the potential of *Bm*-ponericin-L1 as an AMP, further computational studies were performed to predict its atomic coordinates, generating theoretical three-dimensional models. The molecular modeling was carried out using the AlphaFold2 server, resulting in a well-defined α-helical strcuture for *Bm*-ponericin-L1 (Fig. 7a,b). In terms of structure statistics, the lowest free-energy model for*Bm*-ponericin-L1 presents all its amino acid residues in the most favourable regions in the Ramachandran Plot (Supplementary Table S4). Moreover, the overall average of the dihedral angles, along with the backbone’s covalent forces resulted in a G-factor = 0.26 (> − 0.5 for reliable structures) (Supplementary Table S4). As for the physicochemical and antimicrobial prediction calculations, we extended our modeling analysis to the other ponericin-like sequences retrieved from APD. The same modeling methods were used and the theoretical models submitted to stereochemistry and fold quality validations (Supplementary Table S4). Among them, only ponericin-G6 and -G7 showed less than 90% of their amino acid residues in the Ramachandran Plot (Supplementary Table S4). Interestingly, however, when analyzing their secondary structures we observed three different structural profiles, including a set of peptides (including *Bm*-ponericin-L1) with extended α-helix (Fig. 7e), peptides with curved α-helix (Fig. 7f) and peptides presenting α-helix breakage or distorsions triggered by flexibility-inducer residues (*e.g.*, glycine and proline) (Fig. 7g). To the best of our knowledge, this is the first report of the three-dimensional structures of all ponericin-like peptides and their distribution into three structural scaffold “families”.

To improve the accuracy of our in silico studies, structure refinement simulations were performed for *Bm*-ponericin-L1 under two temperatures, 298 and 310 K, thus mimicking the different conditions used in our in vitro experiments (Fig. 7c,d). The refinements were done using MD simulations during 40 ns. As observed in Fig. 7, the refined structures are similar and tend to unfold at the C-terminal region due to the presence of a glycine residue at position 22. As a result, the refined models for *Bm*-ponericin-L1, at both temperatures, present 16.6% less α-helical content than the non-refined structure. Moreover, the root mean square deviation (RMSD) between the *Bm*-ponericin-L1 refined structures is 0.863 Å. When this same analysis is done for the *Bm*-ponericin-L1 non-refined structure and its refined counterparts, a 1.4 Å overall RMSD is observed. In summary, our computational studies indicate that *Bm*-ponericin-L1 presents a well-defined α-helical structural profile with a flexible C-terminus when analysed under 0.15 mol L^−1^ NaCl ionic strength and 298 and 310 K.

### Antibacterial and antibiofilm activity

**Figure 8:**
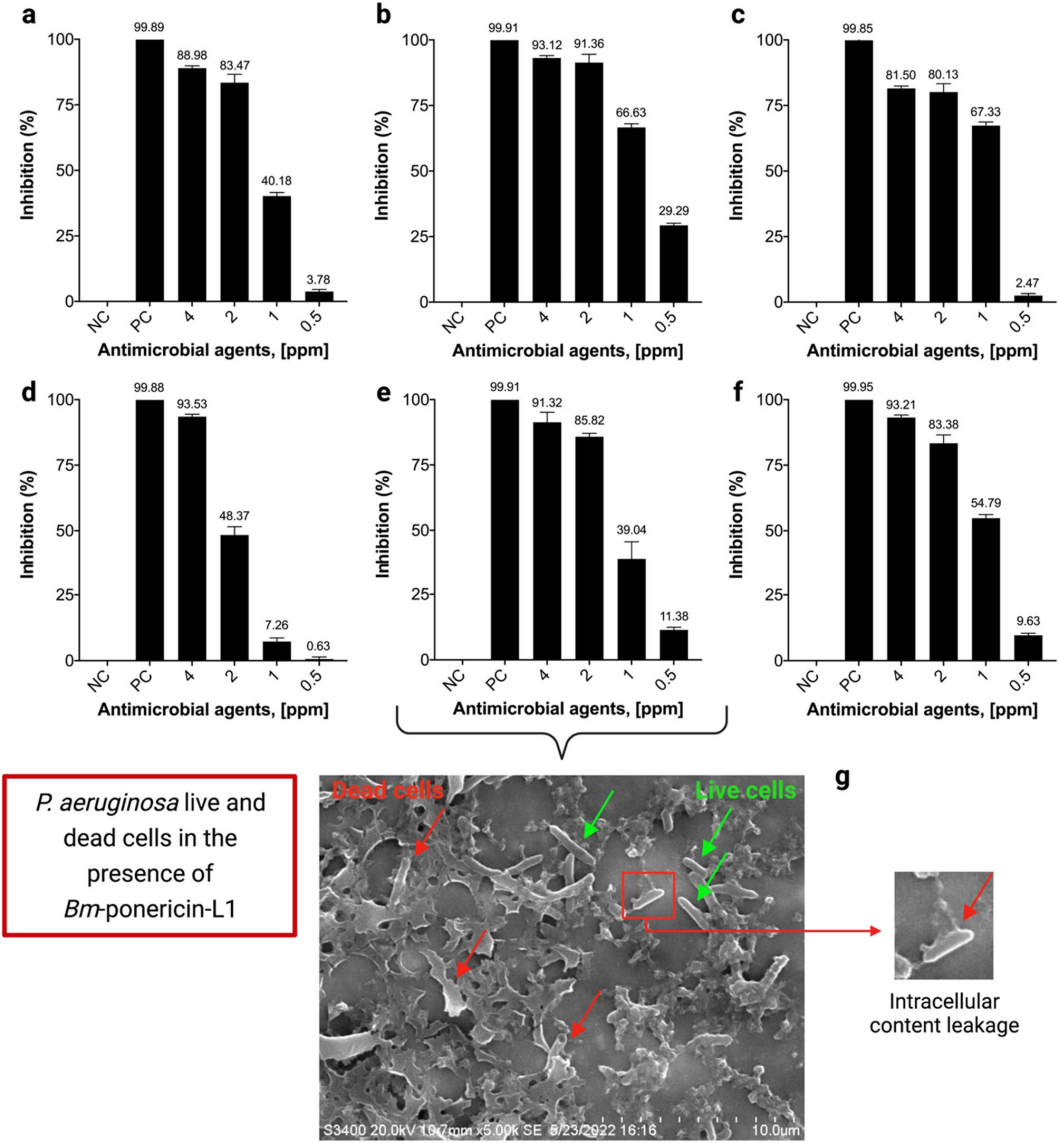
Effect of different concentration of *Bm*-ponericin-L1 on ESKAPE pathogens. (**a**) *E. faecium* (ATCC 35667), (**b**) *S. aureus* (ATCC 6538), (**c**) *K. pneumoniae* (ATCC 70063), (**d**) *A. baumannii* (ATCC 17978), (**e**) *P. aeruginosa* (ATCC 10145), and (**f**) *E. agglomerans* (ATCC 27985). (**g**) SEM micrograph of intact/live bacteria (green arrows) and lysed/dead bacteria (red arrows) in the presence of *Bm*-ponericin-L1. Negative control (NC) and positive control (PC). The data were expressed as mean ± standard deviation and the significance level was set at *p* < 0.05.

The antibacterial activity of *Bm*-ponericin-L1was determined against ESKAPE pathogens and revealed a dose-dependent relationship. The results showed that *Bm*-ponericin-L1 can inhibit the growth of ESKAPE pathogens when compared to the bacterial growth in the negative control. The MIC (IC_50_) of *Bm*-ponericin-L1 ranged from 0.5 to 2 ppm for *E. faecium, S. aureus, K. pneumoniae, P. aeruginosa*, and *E. agglomerans* strains (Fig. 8a–c, e, and f); whereas, > 2 ppm was the MIC (IC_50_) for *A. baumannii* (Fig. 8d). Our study further revealed that there is no strain-dependent variation in the antimicrobial activity of *Bm*-ponericin-L1 when tested against *P. aeruginosa* (ATCC 10145) and *P. aeruginosa* (ATCC 25,668). SEM analysis was performed to evaluate the bactericidal effects of *Bm*-ponericin-L1 on *P.aeruginosa* surface*.* Figure 8g shows the intact morphology with rod-shaped surfaces, as well ascell membrane lysis caused by *Bm*-ponericin-L1 treatment. The result indicates that *Bm*-ponericin-L1 possibly affects intracellular metabolic reactions, causing induction in cytoplasmic damage or leakage (as highlighted). A similar finding was reported earlier for Gram-positive and Gram-negative bacteria treated with an defensin-like AMP derived from oyster^51^.

**Figure 9:**
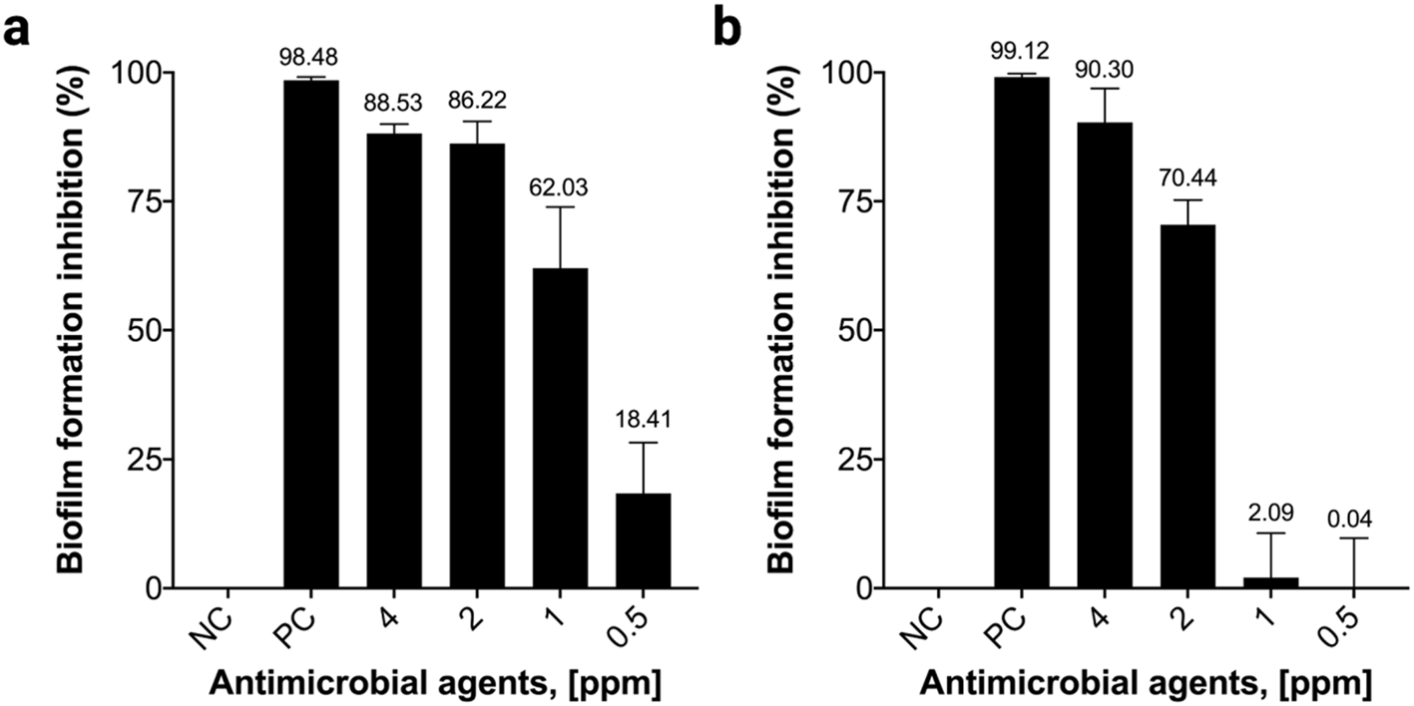
Antibiofilm activity of *Bm*-ponericin-L1 against (**a**) *P. aeruginosa* (ATCC 25668), and (**b**) *K. pneumoniae* (ATCC 70063). The data were expressed as mean ± standard deviation and the significance level was set at *p* < 0.05. NC: negative control. PC: positive control.

In vitro antibiofilm activity of *Bm*-ponericin-L1 was measured at different concentrations against the biofilm-forming bacteria *P.aeruginosa* and *K.pneumoniae.* Our results showed that *Bm*-ponericin-L1, at 4 ppm, inhibited biofilm formation by > 85% (*P. aeruginosa*) and > 90% (*K. pneumoniae*), after 24 h of incubation (Fig. 9a,b). Ourresult is comparable with an earlier study that reported antibiofilm activity of the insect-derived AMP (cecropin A) against uropathogenic *Escherichia coli*^52^.

### Hemolytic and cytocompatibility assays

**Figure 10:**
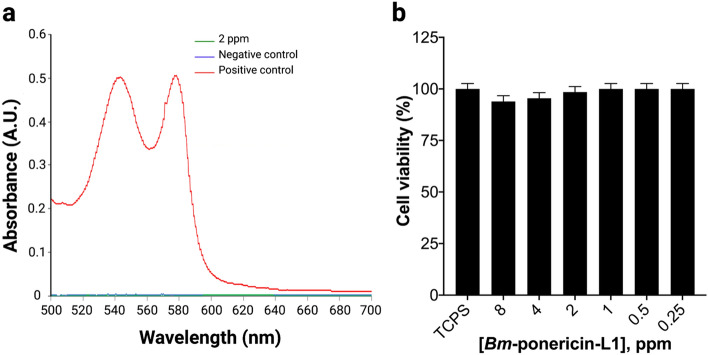
Hemocompatibility and cytocompatibility assay of the purified *Bm*-ponericin-L1 from infected *B. mori* larvae on erythrocytes (**a**) and primary fibroblast cell lines (ATCC PCS-201-012) (**b**) respectively. The data were expressed as mean ± standard deviation and the significance level was set at *p* < 0.05.

Hemolysis is a basic parameter to establish hemocompatibility of antimicrobials^53^. Our results revealed that *Bm*-ponericin-L1 exhibited 0.2% hemolysis (Fig. 10a). It has been reported that antimicrobials with < 5% hemolysis were regarded as hemocompatible^54^. Thus, the *Bm*-ponericin-L1 peptide, at 2 ppm, was found to be more hemocompatiblethan other AMPs reported previously and that have shown hemolyisis in a dose-dependant manner^49^. For this assay, diluted RBCs suspension mixed with 0.8 mL PBS and 0.8 mL double distilled water were used as negative and positive control, respectively.

To evaluate the effects of the *Bm*-ponericin-L1 on the cell viability of primary fibroblast cell lines (ATCC PCS-201-012), MTT assays were conducted with a range of concentrations of the *Bm*-ponericin-L1 from 0.25 to 8 ppm (Fig. 10b). The assay relies on the mitochondrial respiration system, which calculates the cellular energy capacity of a cell. MTT (yellow dye) is reduced by oxido-reductase and NADPH serve as electron donor producing a blue color formazan. This transformation is only feasible in viable cells; hence, the quantity of formazan is directly proportional to the number of viable cells. In a separate study, it was revealed that the insect-derived AMP cecropinXJ inhibited the growth of Huh-7 cells in dose-dependent manner, after 24 h of incubation. In that study, cecropinXJ significantly inhibited the proliferation of Huh-7 cells with an inhibitory rate of ≤ 40% at 10 ppm^55^. By constrast, *Bm*-ponericin-L1 was non-toxic towards primary fibroblast cell lines (ATCC PCS-201-012) (Fig. 10b). Therefore, *Bm*-ponericin-L1 specifically damage or kill bacterial cells, but is non-toxic towards healthy mammalian cells, as reported for other AMPs^56^.

## Conclusion

The main effector molecules of insects immunity are AMPs. AMPs screened from insects possess broad spectrum of activity with the potential to avoid (less likely) antibiotics resistance mechanism. Our study revealed that infection with *P. aeruginosa* triggers the humoral response in *B. mori* larvae via PO activation followed by melanization of both feces and integument, which in turn accelerates AMP production. The infected larvae showed decrease in both gut-weight and silk-gland-weight due to modulation in feeding behavior. The purified peptide fraction screened from the infected silkoworm found to be active against *E. faecium* (ATCC 35667)*, S. aureus* (ATCC 6538)*, K. pneumoniae* (ATCC 70063)*, A. baumannii* (ATCC 17978), *P. aeruginosa* (ATCC 10145), and *E. agglomerans* (ATCC 27985), belonging to the ESKAPE group. Moreover, from this peptide extract, fraction 6 was further characterized by MALDI. As a result from MASCOT analysis, we identified the peptide sequence LLKELWTKMKGAGKAVLGKIKGLL, which was firstly described as an ant venom-derived ponericin-like peptide. To the best of our knowledge, this is the first report regarding the production and isolation of a ponericin in *B. mori* hemolymph upon *P. aeruginosa* infection. This peptide was predicted as an AMP by six different algorithms, and revealing an amphipathic α-helical structural profile with flexible termini (specially, the C-terminus), which are known features for AMP interaction with bacterial membranes for further cell death. Moreover, *Bm*-ponericin-L1was found to be highly specific towards bacterial cells suggesting its cell selectivity. Therefore, this study opened up an unparallel prospect for screening novel AMPs from *B. mori*.

## Supplementary Information

Supplementary Information.

## Data Availability

The mass spectrometry proteomics data have been deposited to the ProteomeXchange Consortium via the PRIDE partner repository with the dataset identifier PXD032794 (http://www.ebi.ac.uk/pride/archive/projects/PXD032794). Individuals wishing to access the data should send a request to either marlonhenrique6@gmail.com or amitmandal08@gmail.com.
