# Supplementary material for: Antimicrobial potential of a ponericin-like peptide isolated from Bombyx mori L. hemolymph in response to Pseudomonas aeruginosa infection
Source: Sci Rep. 2022 Sep 15;12:15493. doi: 10.1038/s41598-022-19450-8 (PMC9477818; doi:10.1038/s41598-022-19450-8)
Supplement: Supplementary file 1 — Supplementary Information. [file 41598_2022_19450_MOESM1_ESM.docx]

Supporting information

Supplementary Table S1. Spectrum analysis report of *Bm*-ponericin-L1.

**Spectrum Analysis Report**

Date: 01/16/2022 Time: 18:01

FileName: D:\Data\Ahmet Kati\09.08.2021_Maldi_1\09.08.2021\LOW25 MIN\0_C5\1\1SRef\pdata\1\1r

Sequence data:

Unmatched

Intensity Coverage: 0.6 % (420 cnts) Sequence Coverage MS: 100.0%

Sequence Coverage MS/MS: 0.0% pI (isoelectric point): 10.8


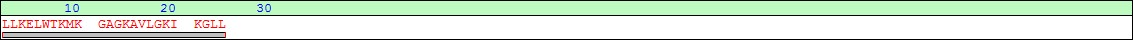
Display Parameter:

Sequence Name: Ponericin-L1 OS=Pachycondyla goeldii PE=1 SV=1 PCL1_PACGO MH+ (mono): 1.008

MH+ (avg): 1.008 Threshold (a.i.): 0.000 Tolerance (Da): 0.500

Number of Peaks: 86

Peaklist:

| **Peak** | **Mass** | **Intensity** | **Peak** | **Mass** | **Intensity** | **Peak** | **Mass** | **Intensity** |
| --- | --- | --- | --- | --- | --- | --- | --- | --- |
| 1 | 598.453 | 184.234 | 2 | 602.298 | 184.007 | 3 | 604.377 | 176.654 |
| 4 | 617.476 | 1998.807 | 5 | 619.483 | 266.369 | 6 | 628.363 | 195.320 |
| 7 | 633.437 | 1113.855 | 8 | 644.128 | 236.982 | 9 | 650.145 | 1018.529 |
| 10 | 655.376 | 225.486 | 11 | 656.134 | 373.600 | 12 | 657.399 | 226.937 |
| 13 | 666.128 | 1038.660 | 14 | 672.124 | 581.174 | 15 | 673.511 | 168.561 |
| 16 | 679.626 | 686.786 | 17 | 682.065 | 343.107 | 18 | 688.112 | 168.160 |
| 19 | 694.273 | 189.167 | 20 | 700.528 | 186.217 | 21 | 761.543 | 687.373 |
| 22 | 762.556 | 276.169 | 23 | 775.468 | 186.379 | 24 | 777.489 | 530.611 |
| 25 | 778.495 | 238.370 | 26 | 781.509 | 172.379 | 27 | 788.676 | 197.136 |
| 28 | 839.133 | 222.851 | 29 | 841.508 | 157.194 | 30 | 855.116 | 243.248 |
| 31 | 861.130 | 549.178 | 32 | 874.620 | 152.781 | 33 | 877.113 | 836.263 |
| 34 | 893.066 | 303.524 | 35 | 905.616 | 230.902 | 36 | 916.631 | 682.168 |
| 37 | 921.620 | 161.355 | 38 | 938.629 | 194.912 | 39 | 1030.689 | 3026.357 |
| 40 | 1037.632 | 136.636 | 41 | 1042.670 | 240.412 | 42 | 1052.670 | 790.199 |
| 43 | 1056.694 | 144.520 | 44 | 1060.672 | 141.457 | 45 | 1064.671 | 330.398 |
| 46 | 1068.654 | 353.645 | 47 | 1090.690 | 145.186 | 48 | 1096.705 | 6004.227 |
| 49 | 1118.689 | 1275.695 | 50 | 1134.661 | 530.373 | 51 | 1359.771 | 109.977 |
| 52 | 1462.901 | 1266.907 | 53 | 1463.396 | 1313.894 | 54 | 1463.840 | 264.288 |
| 55 | 1465.377 | 130.660 | 56 | 1784.069 | 771.114 | 57 | 1806.052 | 187.126 |
| 58 | 1942.118 | 106.962 | 59 | 2072.345 | 455.945 | 60 | 2086.365 | 735.087 |
| 61 | 2094.327 | 182.780 | 62 | 2108.341 | 385.794 | 63 | 2124.279 | 191.217 |
| 64 | 2470.375 | 109.909 | 65 | 2481.458 | 113.244 | 66 | 2502.491 | 154.772 |
| 67 | 2577.543 | 303.048 | 68 | 2595.553 | 545.337 | 69 | 2597.530 | 203.852 |
| 70 | 2682.594 | 474.576 | 71 | 2739.547 | 188.183 | 72 | 2744.728 | 193.882 |
| 73 | 2766.750 | 186.064 | 74 | 2768.541 | 140.984 | 75 | 2888.641 | 415.007 |
| 76 | 2891.686 | 622.266 | 77 | 2896.657 | 324.609 | 78 | 2900.361 | 472.982 |
| 79 | 2907.839 | 654.826 | 80 | 2910.958 | 2996.215 | 81 | 2916.626 | 326.597 |
| 82 | 2920.730 | 399.996 | 83 | 2924.769 | 21695.772 | 84 | 2939.852 | 449.620 |
| 85 | 2946.768 | 492.606 | 86 | 2980.816 | 445.634 |  |  |  |

| Supplementary Table S2 – Physicochemical properties for all 16 ponericin-like peptides deposited in APD. | | | | |
| --- | --- | --- | --- | --- |
| Peptide name | **Sequence** | **Charge (z)** | **Hydrophobicity (%)** | **Hydrophobic moment (<μH>)** |
| Ponericin-G1 | _1_GWKDWAKKAGGWLKKKGPGMAKAALKAAMQ_30_ | +7 | 22.0 | 0.228 |
| Ponericin-G2 | _1_GWKDWLKKGKEWLKAKGPGIVKAALQAATQ_30_ | +5 | 28.7 | 0.331 |
| Ponericin-G3 | _1_GWKDWLNKGKEWLKKKGPGIMKAALKAATQ_30_ | +6 | 23.2 | 0.366 |
| Ponericin-G4 | _1_DFKDWMKTAGEWLKKKGPGILKAAMAAAT_29_ | +3 | 30.8 | 0.347 |
| Ponericin-G5 | _1_GLKDWVKIAGGWLKKKGPGILKAAMAAATQ_30_ | +5 | 38.5 | 0.290 |
| Ponericin-G6 | _1_GLVDVLGKVGGLIKKLLP_18_ | +2 | 60.8 | 0.587 |
| Ponericin-G7 | _1_GLVDVLGKVGGLIKKLLPG_19_ | +2 | 57.6 | 0.556 |
| Ponericin-L1* | _1_LLKELWTKMKGAGKAVLGKIKGLL_24_ | +5 | 45.8 | 0.542 |
| Ponericin-L2 | _1_LLKELWTKIKGAGKAVLGKIKGLL_24_ | +5 | 48.2 | 0.566 |
| Ponericin-W1 | _1_WLGSALKIGAKLLPSVVGLFKKKKQ_25_ | +6 | 47.5 | 0.403 |
| Ponericin-W2 | _1_WLGSALKIGAKLLPSVVGLFQKKKK_25_ | +6 | 47.5 | 0.413 |
| Ponericin-W3 | _1_GIWGTLAKIGIKAVPRVISMLKKKKQ_26_ | +7 | 42.0 | 0.365 |
| Ponericin-W4 | _1_GIWGTALKWGVKLLPKLVGMAQTKKQ_26_ | +5 | 50.9 | 0.305 |
| Ponericin-W5 | _1_FWGALIKGAAKLIPSVVGLFKKKQ_24_ | +5 | 55.9 | 0.497 |
| Ponericin-W6 | _1_FIGTALGIASAIPAIVKLFK_20_ | +2 | 78.0 | 0.403 |
| Ponericin-Q42 | _1_FWGAVWKILSKVLPHIPGTVKWLQEKV_27_ | +3 | 71.9 | 0.531 |
| APD: Antimicrobial Peptide Database. Hydrophobic moment<μH> calculated according to the Eisenberg scale. The physicochemical properties were calculated on HeliQuest server (https://heliquest.ipmc.cnrs.fr).*Corresponds to ponericin-L1, the same peptide sequence identified in our work, named *Bm*-ponericin-L1. | | | | |

| Supplementary Table S3 – Antimicrobial predictions for all 16 ponericin-like peptides deposited in APD. | | | | | | |
| --- | --- | --- | --- | --- | --- | --- |
| Peptide name | **SVM** | **RF** | **ANN** | **DA** | **DBAASP** | **STM** |
| Ponericin-G1 | 0.996 | 0.962 | AMP | 0.999 | AMP | 0.648 |
| Ponericin-G2 | 0.994 | 0.998 | AMP | 0.999 | AMP | 0.685 |
| Ponericin-G3 | 0.993 | 0.996 | AMP | 0.998 | AMP | 0.798 |
| Ponericin-G4 | 0.752 | 0.817 | NAMP | 0.970 | AMP | 0.822 |
| Ponericin-G5 | 0.992 | 0.995 | AMP | 0.999 | AMP | 0.647 |
| Ponericin-G6 | 0.945 | 0.874 | AMP | 0.997 | AMP | 1.116 |
| Ponericin-G7 | 0.958 | 0.968 | AMP | 0.998 | AMP | 1.052 |
| Ponericin-L1* | 0.991 | 0.998 | AMP | 0.993 | AMP | 0.881 |
| Ponericin-L2 | 0.993 | 0.999 | AMP | 0.999 | AMP | 0.942 |
| Ponericin-W1 | 0.989 | 1 | AMP | 1 | AMP | 0.788 |
| Ponericin-W2 | 0.986 | 1 | AMP | 1 | AMP | 0.796 |
| Ponericin-W3 | 0.995 | 1 | AMP | 0.999 | AMP | 0.821 |
| Ponericin-W4 | 0.992 | 0.996 | AMP | 0.999 | AMP | 0.629 |
| Ponericin-W5 | 0.998 | 1 | AMP | 1 | AMP | 0.726 |
| Ponericin-W6 | 0.991 | 0.962 | AMP | 0.999 | NAMP | 0.645 |
| Ponericin-Q42 | 0.881 | 0.917 | AMP | 0.939 | AMP | 0.944 |
| APD: Antimicrobial Peptide Database; SVM: Support Vector Machine; RF: Random Forest; ANN: Artificial Neural Network; DA: Discriminant Analysis; DBAASP: Database of Antimicrobial Activity and Structure of Peptides; STM: Sense The Moment. Probabilities and scores higher than 0.5 indicate positive prediction for antimicrobial activity (AMP). *Corresponds to ponericin-L1, the same peptide sequence identified in our work, named *Bm*-ponericin-L1. | | | | | | |

| Supplementary Table S4 – Structural statistics for three-dimensional theoretical models for all 16 ponericin-like peptides deposited in APD. | | | | | | |
| --- | --- | --- | --- | --- | --- | --- |
| Peptide name | **APD ID** | **Ramachandran (most favored) (%)** | **G-factors** | **Z-score** | **Bad bonds (%)** | **Bad angles (%)** |
| Ponericin-G1 | AP00376 | 95.7 | -0.25 | -2.33 | 3.46 | 0.33 |
| Ponericin-G2 | AP00377 | 95.8 | -0.12 | -1.94 | 2.92 | 0.93 |
| Ponericin-G3 | AP00378 | 95.8 | -0.12 | -2.0 | 3.28 | 1.23 |
| Ponericin-G4 | AP00379 | 95.8 | -0.15 | -2.0 | 3.28 | 1.23 |
| Ponericin-G5 | AP00380 | 95.7 | -0.23 | -1.3 | 4.05 | 1.69 |
| Ponericin-G6 | AP00381 | 84.6 | -0.14 | 0.33 | 1.57 | 2.37 |
| Ponericin-G7 | AP00382 | 84.6 | -0.5 | 0.4 | 2.29 | 1.72 |
| Ponericin-L1* | AP00383 | 100 | 0.26 | -1.71 | 0.55 | 0.89 |
| Ponericin-L2 | AP00384 | 100 | 0.36 | -1.44 | 0.55 | 1.67 |
| Ponericin-W1 | AP00386 | 100 | -0.23 | -1.2 | 4.12 | 1.56 |
| Ponericin-W2 | AP00387 | 100 | -0.27 | -1.24 | 5.15 | 1.17 |
| Ponericin-W3 | AP00388 | 95.2 | -0.10 | -0.58 | 1.98 | 0.75 |
| Ponericin-W4 | AP00389 | 95 | -0.19 | -0.88 | 3.43 | 1.10 |
| Ponericin-W5 | AP00390 | 100 | -0.16 | -1.27 | 4.79 | 1.20 |
| Ponericin-W6 | AP00391 | 100 | 0.05 | -1.6 | 1.38 | 2.05 |
| Ponericin-Q42 | AP02435 | 95.2 | -0.01 | -0.44 | 2.14 | 0.94 |
| The z-scores obtained for all structures here reported are in agreement with those with similar size, structurally determined by X-ray crystallography and deposited in the Protein Data Bank (PDB). The G-factors indicate that the overall average for the dihedral angles, along with the main-chain covalent forces for each structure are within the expected values for reliable structures (G-factors > −0.5). The structural validations were performed on PROCHECK, ProSa-web, PROCHECK and MolProbity. *Corresponds to ponericin-L1, the same peptide sequence identified in our work, named *Bm*-ponericin-L1. | | | | | | |


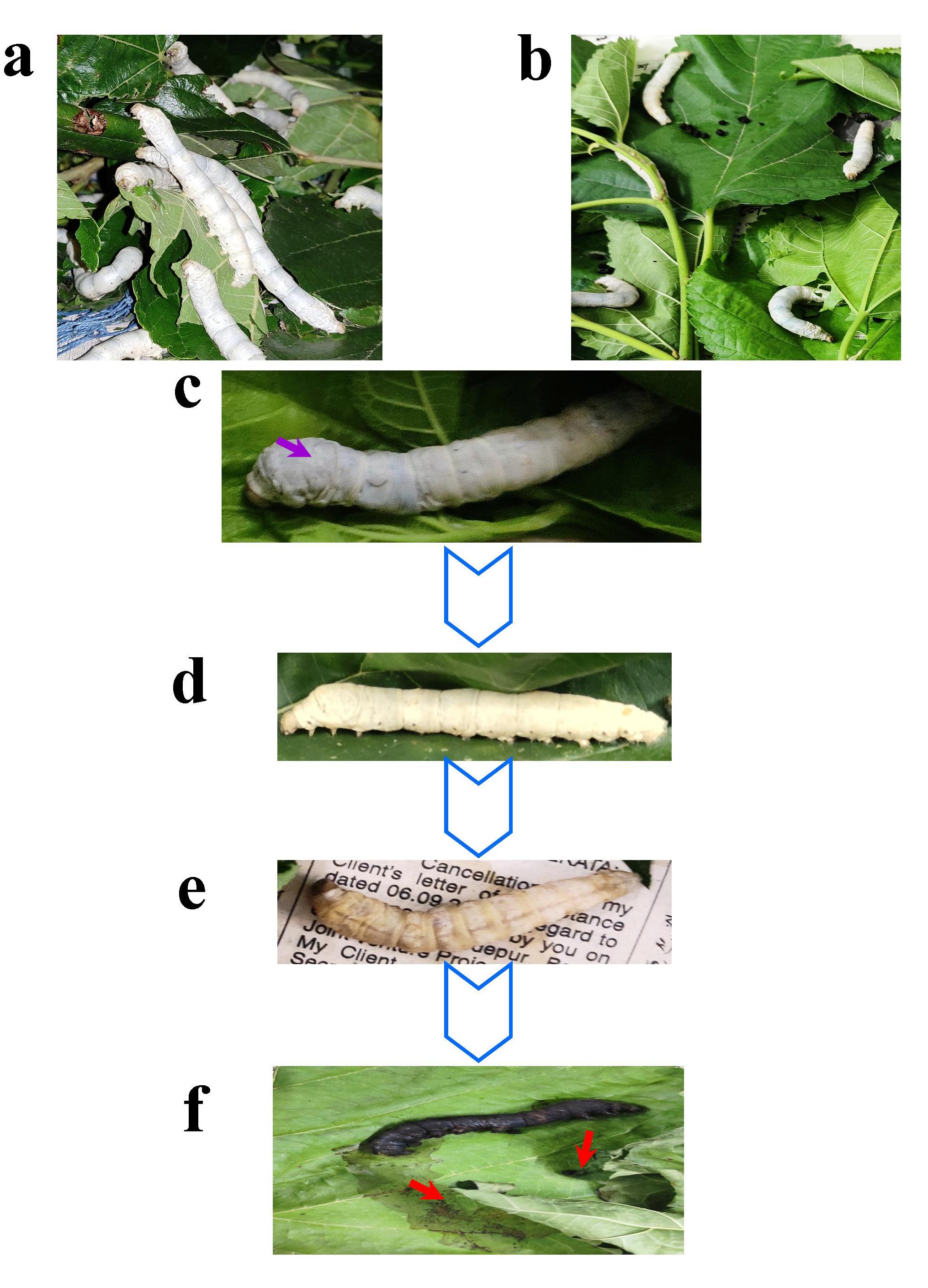


Supplementary Figure S1.Modulation in foraging behavior and integument in non-immunized and immunized *B. mori* larvae. (a) Continued feeding of untreated larvae. (b) Discontinued feeding in immunized larvae. (c) Purple color arrow showing the swelling in head and thorax. (d-f) Changes in larval color from white to pale yellow, coffee brown and, ultimately, black due to onset of infection. Hemolymph discharged from ruptured integument indicated by red color arrow.


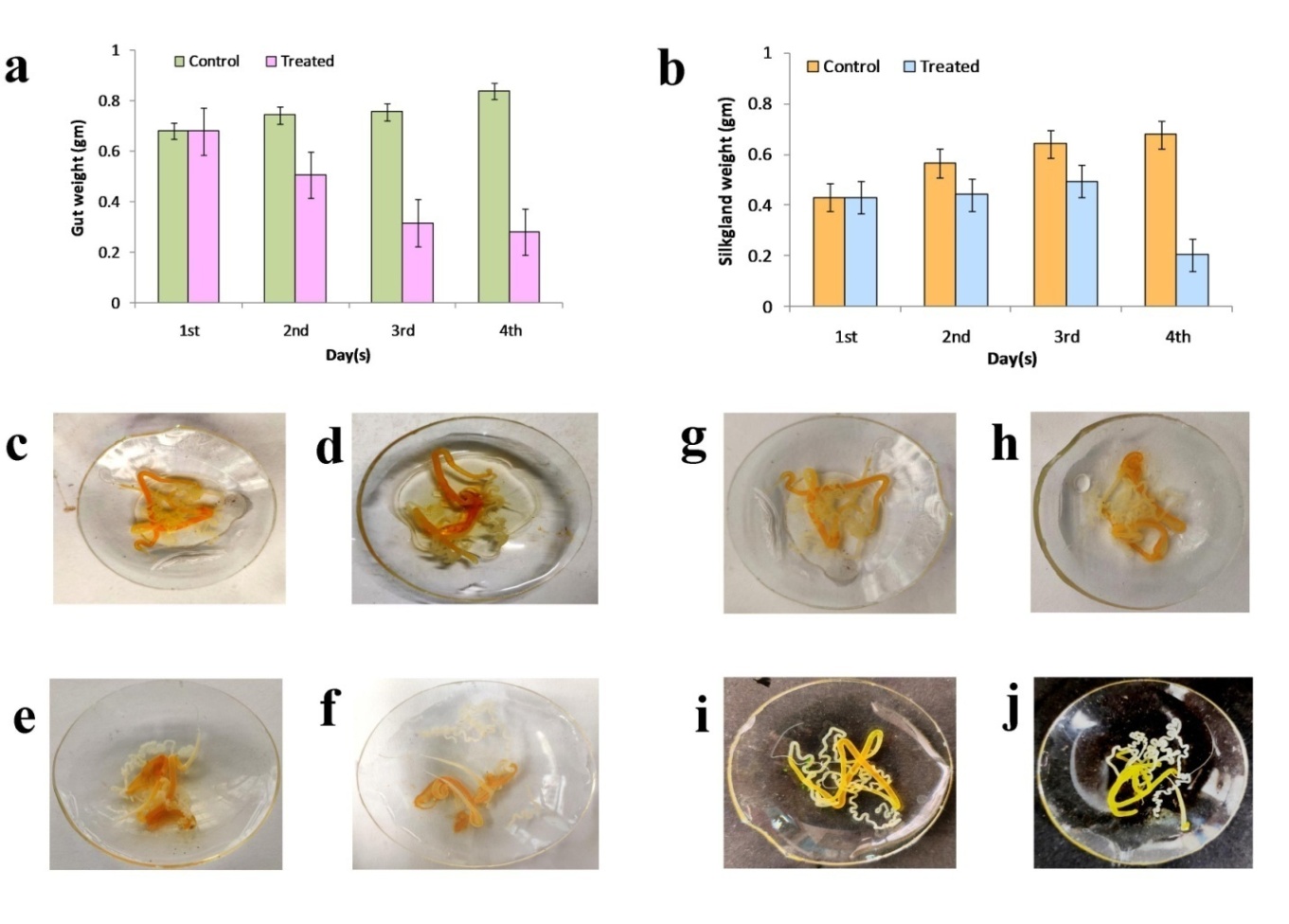


Supplementary Figure S2.Changes in gut weight and silk gland weight in immunized and non-immunized *B. mori* larvae. Changes in silk gland color in immunized and non-immunized silkworms. The loss of craving activity triggered immunized silkworm causes significant decrease in both wet weight of gut and wet weight of the silk gland compared to the control group (a & b). Decrease in silk production resulting in white color silk gland (c-f). The silk gland of the dissected non-immunized group showed a gradual increase in the intensity of yellow color, which is probably due to normal feeding behavior accompanied by increased silk production (g-j).
